# Health benefits of decarbonization and clean air policies in Beijing and China

**DOI:** 10.1088/1748-9326/ad8c65

**Published:** 2024-11-15

**Authors:** Gregor Kiesewetter, Shaohui Zhang, Jun Liu

**Affiliations:** 1 Energy, Climate, and Environment Program, International Institute for Applied Systems Analysis, Schlossplatz 1, 2361 Laxenburg, Austria; 2 Department of Environmental Engineering, School of Energy and Environmental Engineering, University of Science and Technology Beijing, Beijing 100083, People’s Republic of China

**Keywords:** integrated assessment modelling, air pollution, health co-benefits, GAINS model, decarbonization

## Abstract

Although China has seen strong reductions in air pollution levels in the last decade, PM_2.5_ concentrations still exceed the WHO Guideline several times, causing a substantial burden of mortality and morbidity. With many ‘low hanging fruits’ in terms of abatement measures already taken, further improvements will be more difficult and likely require different strategies than pursued so far. This study looks into the trends expected under current energy policies and air pollution control legislation and analyses the source contributions to ambient PM_2.5_ in China, with a special focus on the megacity of Beijing. Although reductions are foreseen, China appears not yet on track to meet its long-term targets for greenhouse gas emissions nor the future national air quality standards. Going beyond current policies, we analyze effects of measures which tackle both issues and quantify health co-benefits from further decarbonization policies required to meet the national target of reaching carbon neutrality by 2060, as well as the potential for further air pollution mitigation.

## 1. Introduction

After years of record high air pollution episodes, China has managed strong and unprecedented reductions of ambient PM_2.5_ concentrations after 2013. The reductions came as a result of strong policy interventions which targeted first SO_2_ and later NO_x_ emissions from power and industry, as well as coal in residential combustion. Reductions of PM_2.5_ emissions from large point sources were achieved already earlier through mandatory installation of cyclone or electrostatic precipitators.

In particular the region around Beijing and its neighboring provinces Tianjin and Hebei, known as Jing-Jin-Ji, was a global air pollution hot spot in the 2000s. PM_2.5_ concentration levels in Beijing have dropped from an annual average of more than 81 *µ*gm^−3^ in 2015 to 33 *µ*gm^−3^ in 2022 (Zhang *et al* 2023). In Beijing specifically, part of the reduction was achieved by relocation of heavy industries outside province boundaries in conjunction with consolidation of capacity and phase-out of small industrial production facilities (Zhang *et al* 2019). Significant progress was also made through elimination of coal for heating in the city and replacement by piped gas, district heating, and electricity. A similarly aggressive policy on fuel switching out of coal in urban areas has been implemented in the neighboring provinces as well, though consumption of coal in the residential sector is still significant in Hebei, mostly in rural settings which cannot be easily connected to the gas or district heating networks (Meng *et al* 2019).

Despite the progress made in the last decade, however, annual mean PM_2.5_ concentration levels still exceed the WHO 2021 guideline level of 5 *µ*gm^−3^ by more than a factor of 5, and the national standard of 35 *µ*g m^−3^ is not yet achieved everywhere either. The question thus arises whether the fast progress in air quality improvement can continue given that many of the ‘low-hanging fruits’ in terms of emission controls have been reaped. The year of 2023 and the winter of 2023–2024 has seen higher concentrations than the year before, and several cities in Eastern China missed their ambient pollution reduction targets set under the annual Winter action plan (MEE of China 2024), clearly showing that the battle for clean air is not yet won and continuous enforcement and tightening of policies will be needed in order to improve the situation further.

Independent of the targets for air quality, China has also committed to achieve carbon neutrality in 2060. Given that many sources of greenhouse gas emissions are also sources for air pollution, there is potential for synergies to achieve both targets (Zhang *et al* 2021). PM_2.5_ is putting a high health burden on the Chinese population with an estimated number of 1.2 million premature deaths annually (Zhang *et al* 2023). If implemented well, measures towards greenhouse gas (GHG) reductions can thus have significant co-benefits on public health. Several studies have investigated this for China: (Zhao *et al* 2024) evaluated the Chinese historical air pollutant emissions and the associated drivers under provincial and sector scales. They found that end-of-pipe technologies have been essential for pollutant abatement, especially for the power and industry sector. Further improvement of air quality would require integrated solutions and regional cooperation, due to the influence of transboundary effects and the decreased potential of air pollution abatement (Jiang *et al* 2023). Cheng *et al* (2023) used an integrated assessment model to explore potential air quality improvements and associated health effects under different decarbonization scenarios in key regions of China (e.g. Beijing-Tianjin-Hebei) (Cheng *et al* 2023). They found that around 1 million annual premature deaths would be avoided in China by 2060 under the carbon zero actions and air pollution control measures. However, state-of-the-art studies rarely quantify the potential air quality improvements for megacities and the transboundary contributions under various climate change mitigation measures and end-of-pipe control options.

In this study we quantify the health implications of pathways for simultaneously achieving the decarbonization and clean air targets. We employ the Greenhouse Gas—Air Pollution Interactions and Synergies (GAINS) model (Amann *et al* 2020) to analyze different scenarios in terms of energy pathways as well as air pollution emission control legislation, quantifying the effects of sectoral measures. The analysis is done for the whole of China, with an additional focus on Beijing as an example of a megacity which has already achieved strong reductions in pollution levels but is now facing the question how to continue towards clean air when most local sources are already controlled.

Going beyond previous analysis, besides analyzing contrasting energy pathways, we use the granular representation of air pollution control technologies in GAINS to develop different sets of control strategies reaching from current legislation to full exploitation of the potential in all sectors. We quantify the potential effects and benefits of packages of measures in order to identify priority areas for achieving synergies between climate and clean air policies both for Beijing (with a particular focus on the inflow of pollution from surrounding provinces) as well as for other regions in China.

This manuscript is organized as follows. The models used and scenarios developed are introduced in section 2. Section 3 presents the results in terms of modelled PM_2.5_ concentrations and health impacts for the different scenarios. Estimates of source contributions from different spatial origins, sectors and fuels are given, and their likely evolution under different scenarios illustrated.

## 2. Methodology

To explore the conceivable range of future ambient PM_2.5_ concentrations, this study develops a series of alternative emission scenarios up to 2050. The scenarios combine different assumptions on the key policy areas that have been identified as critical for air pollution trends in the past, i.e. (a) energy/climate policy, (b) agricultural policies, and (c) dedicated emission control policies (Rafaj *et al* 2014). We apply the GAINS model (East Asia version^3^3Online at https://gains.iiasa.ac.at/gains/EAN/index.login?logout=1 (user registration required)., release 4.0.3) to quantify emissions of air pollutants for each province by combining projections of activity levels with assumptions on emission control legislation and information about emission factors of individual control technologies. GAINS is an established integrated assessment model to analyze air pollution emissions and impacts under different policy scenarios, it has been applied to analysis in China before (Li *et al* 2019, Liu *et al* 2019, Zhang *et al* 2021). A detailed description of the GAINS model can be found in the SI (supplementary text A1.1). The resulting emission trends are fed into a simplified atmospheric chemistry and transport model to compute ambient concentrations of PM_2.5_ and the contributions from different sectors and source regions (details in supplementary text A1.2). Premature mortality attributable to long-term exposure to PM_2.5_ is calculated following the Global Burden of Disease approach, using the MR-BRT concentration-response functions developed for GBD2019 (GBD Collaborative Network 2021) (details in supplementary text 1.3).

### 2.1. Linkage between energy models and GAINS

GAINS relies on external inputs for activity projections, which in our study come from the MESSAGEix model (Liu *et al* 2022) and GCAM-China (Xing *et al* 2020). A soft linking approach is utilized to establish the linkage between the energy models and the GAINS model, allowing for seamless integration and data exchange. In this process, provincial-level activity data for various sectors such as power, transportation, industry, agriculture, and household are reported and harmonized to be integrated into the GAINS model. More information on the steps is given in the SI (supplementary text A1.2).

### 2.2. Scenarios

The main elements distinguishing scenarios are the activity projections (energy and agriculture pathways) and the assumptions on control technologies applied.

Energy pathways for China outside Beijing have been developed with the GCAM-China model. GCAM-China, an integrated assessment model, has been widely used to estimate energy supplies, demands, technology adoption, fuel use, and GHG emissions, at the provincial level. In this study, the base year of GCAM-China is 2010, running 5 year steps up to 2100, and the calibrations are conducted for 2015 and 2020. Projections up to 2050 have been imported into GAINS. In order to allow for a zoom into Beijing, we use for this province energy projections which have been generated with the MESSAGEix provincial energy system model and published before by Liu *et al* (2022).

We analyze a Baseline case, which follows business as usual, and a decarbonization case. The Baseline scenario considers energy policies which are already in place or firmly committed. It includes the implications of the 13th five year plan and achieves the Nationally Determined Contributions (NDCs) specified by China for 2030 (UNFCCC 2021), but it does not assume further far-reaching transitions away from coal. A 2020–2060 CO_2_ emission cap from Fawcett *et al* (2015) is applied. CO_2_ emissions peak at around 12 Gt in 2030, followed by 1%–2.5% reduction per year after 2030.

In contrast to the Baseline, the Net-Zero (NZE) pathway achieves carbon neutrality in Beijing in 2050 and in the rest of China by 2060. Electrification is the key element for decarbonization. In Beijing, we use the IND (indigenous production) scenario from Liu *et al* (2022) which assumes increased electricity generation within Beijing’s province boundaries through gas power stations coupled with carbon capture and storage to offset the resulting CO_2_ emissions. In the rest of China, for the NZE pathway, the key assumption is in line with the dual carbon goals, i.e. the latest submitted NDC and carbon neutrality by 2060.

The scenarios include projections for agricultural production from the GCAM-China model. The net-zero pathway does not include assumptions on dietary changes, therefore the agricultural projections are identical between Baseline and NZE. Other non-energy sectors (like waste and wastewater) are based on GAINS internal projections (Gomez Sanabria *et al* 2021).

Emissions of air pollutants are not only determined by the activity levels and amounts of fuels consumed but also by the end of pipe emission controls applied. The GAINS model allows to explore the effects of emission control legislation, independent of the activity pathway chosen. We investigate two different scenarios here:

• In the Current Legislation case (CLE), the emission control measures applied in the GAINS model reflect our best understanding of current regulations and practices in each sector. It includes all policies and measures that are already implemented today or have been announced. For those that have been announced, the extent and timing of their implementation is assessed according to the prevailing institutional, political and economic circumstances.
• As an alternative to the CLE, the Maximum Technically Feasible Reduction (MFR) case assumes complete implementation of the best currently available technologies to the extent feasible in a given year in the future. The potential is limited by stock turnover and capacity exchange but not by costs.

An overview of the combined scenarios is given in table 1. They are constructed such that we can explicitly distinguish the effects of decarbonization policies in Beijing alone, decarbonization in surrounding provinces, and end-of-pipe air pollution control measures.

**Table 1.** Composition of scenarios analyzed in this study. Abbreviations: IND = Decarbonization in Beijing via indigenous electricity production (MESSAGEix model), NZE = Net-zero (GCAM model), CLE = Current legislation, MFR = Maximum technically feasible reductions.

Scenario	Energy Beijing	Energy other provinces	Air pollution emission controls
Baseline	Baseline	Baseline	CLE
IND	IND	Baseline	CLE
IND_NZE_CLE	IND	NZE	CLE
IND_NZE_MFR	IND	NZE	MFR

## 3. Results

In this section we present ambient concentrations of PM_2.5_ and related health impacts estimated with the GAINS model for different scenarios. Section 3.1 discusses emissions and concentration trends for China expected under the different scenarios. Health impacts and contributions from different sectors and fuels are discussed in section 3.2. The effect of individual measures on PM_2.5_ and GHG emissions at national level is quantified in section 3.3. Section 3.4 takes a deeper look at the megacity of Beijing, analyzing local versus transboundary contributions and the effects of individual measures.

### 3.1. Emissions and ambient PM_2.5_ concentrations

**Figure 1.**
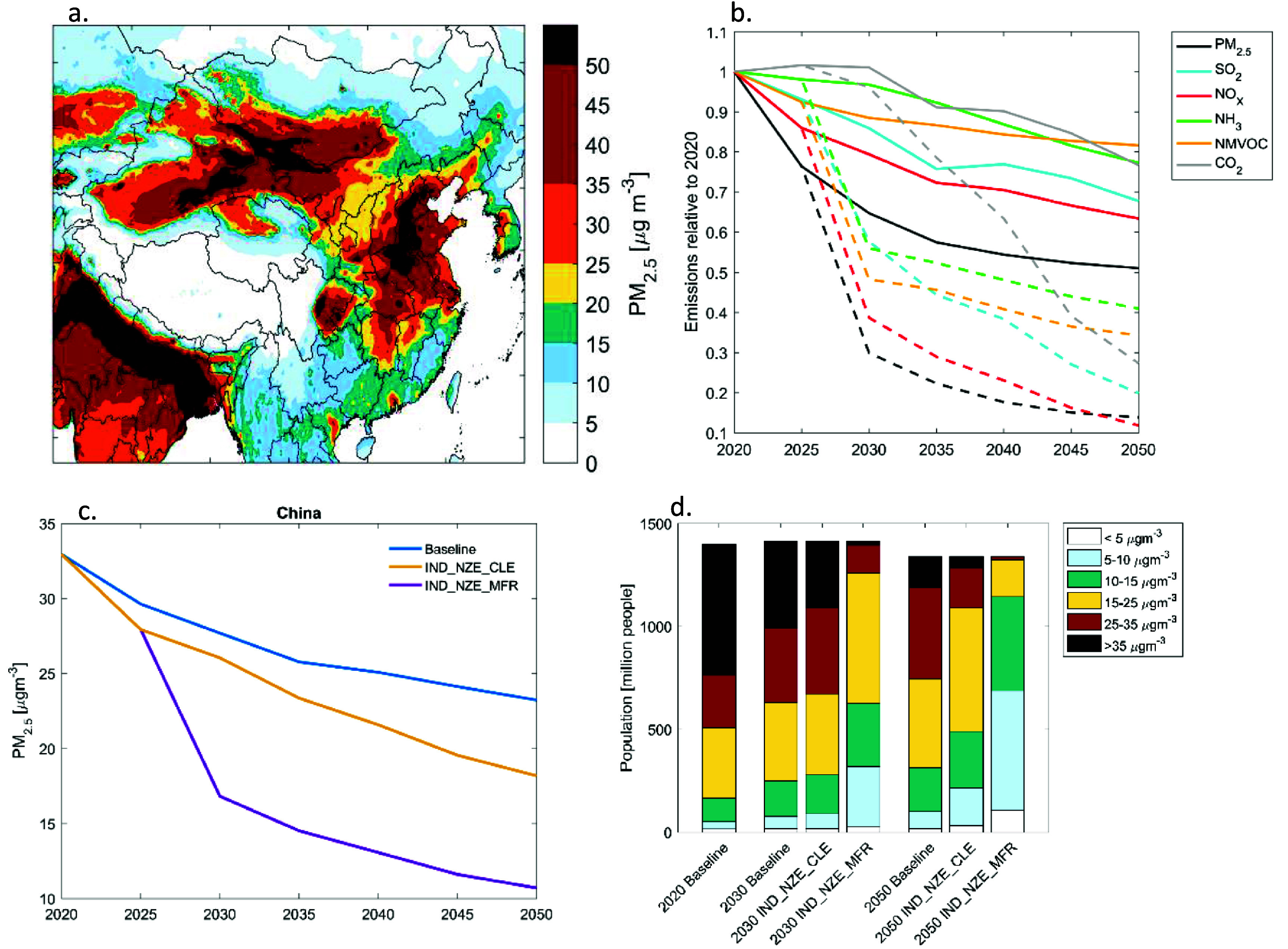
(a) Ambient PM_2.5_ concentrations modelled for 2020; (b) emissions of PM precursor pollutants and CO_2_ in China in the Baseline scenario (solid lines), and in IND_NZE_MFR (dashed lines); (c) annual population-weighted mean ambient PM_2.5_ concentrations in China; (d) population exposure distribution to PM_2.5_ under different scenarios.

Modelled annual mean PM_2.5_ concentrations for 2020 are shown in figure 1(a). Although concentration levels have continuously dropped in the past decade, a substantial part of China, as well as of Beijing province, is still above the National Standard of 35 *µ*gm^−3^ which is at the same time WHO Interim Target 1. A comparison of modeled versus observed annual mean concentrations for individual monitoring stations in 2020 is shown in figure S2 (SI) and for 2015 in figure S3. In 2020, GAINS reproduces measured ambient PM_2.5_ concentrations and their variability in the whole of China (mean bias −0.3 *µ*gm^−3^, *R*^2^ = 0.53) and in Beijing quite well (mean bias +2.9 *µ*gm^−3^, *R*^2^ = 0.50). Annual mean observed concentrations were calculated from hourly PM_2.5_ data from the China Environmental Monitoring Center^4^4source: https://datacenter.mee.gov.cn/websjzx/queryIndex.vm and https://air.cnemc.cn:18007/.; only stations with more than 80% temporal coverage are used in the comparison. These official monitoring stations are in good agreement with the measurements reported by the US embassies in 3 cities (figure S4). Decreases in ambient PM_2.5_ from 2015 to 2020 are reproduced in the model (−8.9 *µ*gm^−3^ population-weighted mean across China) but are smaller than in observations, which may be partly due to constant atmospheric conditions being used in GAINS.

Emission projections of PM precursors in the Baseline scenario are shown in figure 1(b) for the whole of China (numerical values see supplementary table A5 in the SI); an equivalent plot for Beijing is in the SI (figure S5). Further decreases of emissions of all air pollutants are expected, though trends differ for individual pollutants. NH_3_ and VOC emissions are not expected to change much without further legislation. PM_2.5_ and NO_x_ emissions show stronger declines while continued reliance on coal in the Baseline hinders decreases in SO_2_ below 70% of the 2020 level. Sharper decreases before 2035 are partly driven by the Beautiful China Target for 2035; the further evolution of the Baseline suggests that there is a risk of a slight rebound of emissions after this year if no equivalent target is set for later years.

Under the IND_NZE_MFR scenario, the other extreme of the range indicated as dashed lines in figure 1(b), emissions of all pollutants decline steeply, with PM_2.5_, NO_x_ and SO_2_ emissions between 10%–20% of the 2020 values in 2050.

The decreasing emissions lead to decreasing ambient pollution, as shown in figure 1(c) for population-weighted annual mean PM_2.5_ concentrations. At the national level, population weighted mean concentrations in the Baseline scenario are expected to decrease from 33.5 *µ*gm^−3^ in 2020 to 20.8 *µ*gm^−3^ in 2050. Decarbonization in the IND_NZE_CLE reduces concentrations by 5.1 *µ*gm^−3^ in 2050, while additional end-of-pipe controls implemented in IND_NZE_MFR would reduce by additional 8.3 *µ*gm^−3^ to a national average of 10.6 *µ*gm^−3^, out of which 3.2 *µ*gm^−3^ are natural and thus not influenced by the emission changes. Significant variability in concentration levels persists, so that under the Baseline still 11% of China’s population would remain above current air quality standards (35 *µ*gm^−3^), whereas the IND_NZE_MFR brings 99% of the population below the envisaged standard of 25 *µ*gm^−3^ in 2050 (figure 1(d)).

### 3.2. Health benefits of mitigation

**Figure 2.**
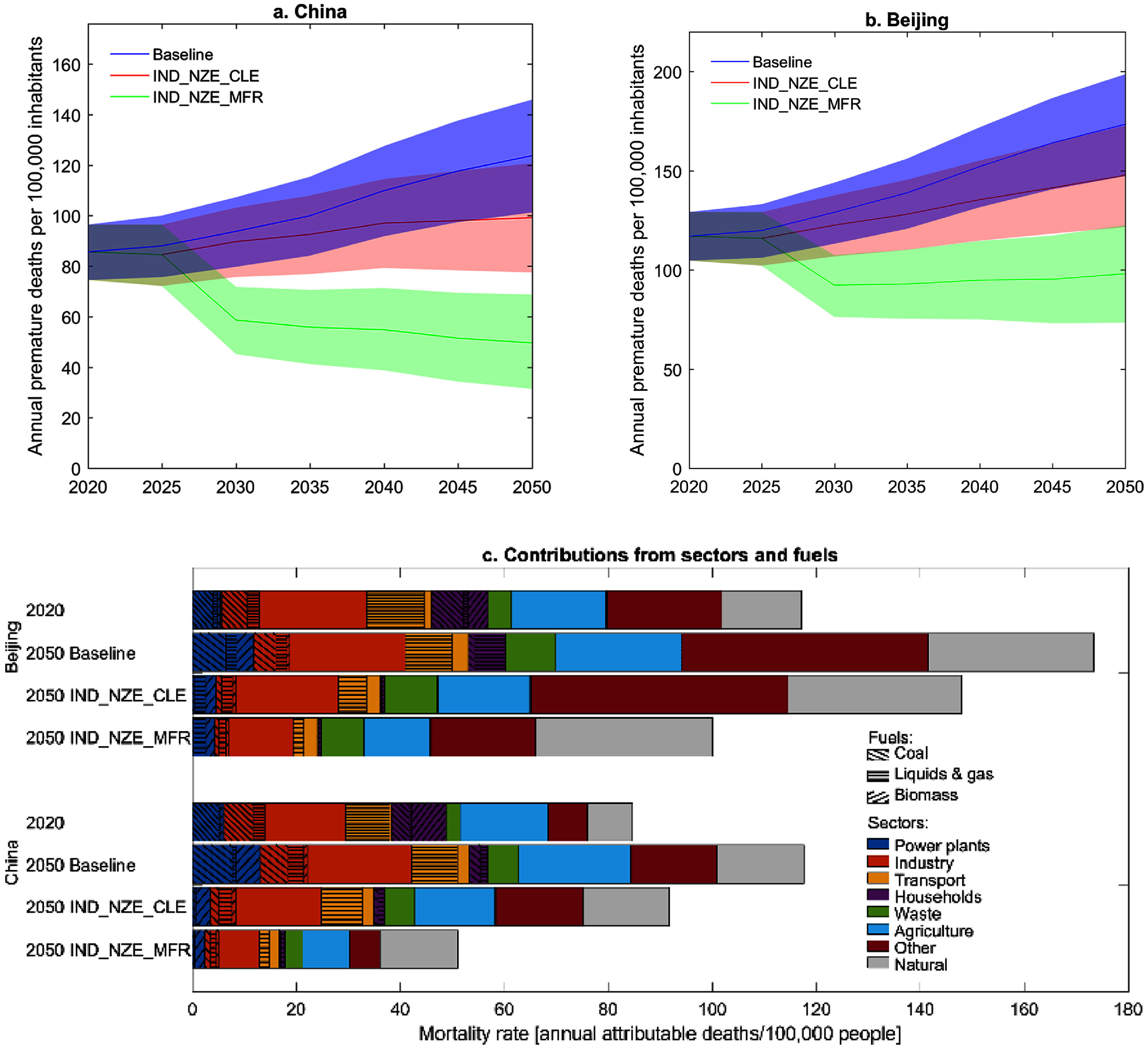
Premature deaths attributable to ambient PM_2.5_ per 100 000 inhabitants in the different scenarios: Trends in China (a) and Beijing (b), and contributions from sectors and fuels in selected scenario years (c) (See table 1 for scenario definition and supplementary table A6 (SI) for numerical values). Shaded areas in (a) and (b) indicate 95% CI ranges.

Changes in ambient PM_2.5_ levels are associated with changes in air pollution related premature mortality. We estimate that in 2020, 1.23 (95% CI 1.07–1.38) million premature deaths in China are attributable to ambient PM_2.5_, out of which 26 600 (23 800–29 400) in Beijing. By 2050, despite decreasing concentration levels in the Baseline, total premature deaths are expected to increase to 1.70 (1.39–2.00) million in China (figure 2(a)) and 46 600 (39 500–53 300) in Beijing (figure 2(b)) due to population aging. Since the largest population cohorts will approach their end of life, baseline death numbers are increasing, corresponding to more attributable deaths at the same time. Similar results have been reported before (Rafaj *et al* 2021). Decarbonization in the IND_NZE_CLE scenario reduces the burden to 1.36 (1.06–1.66) million cases in China, and the additional application of best available end-of-pipe emission abatement technologies across all sectors in the IND_NZE_MFR scenario reduces it further to 679 000 (431 000–945 000) cases per year.

When comparing different sub-national regions, it is useful to normalize to population and show mortality attributable to PM_2.5_ rather than absolute numbers of premature deaths. Although trends are similar in Beijing and China total, absolute mortality per 100 000 inhabitants is higher in Beijing due to the elevated PM_2.5_ concentrations in the area.

Contributions from different sectors and fuels to mortality attributable to ambient PM_2.5_ in Beijing and China are shown in figure 2(c) for 2020 and individual scenarios in 2050 (see supplementary table A6 for numerical values). While fossil fuels still contribute a substantial share of ambient PM_2.5_ and related mortality in 2050 under the Baseline scenario (403 000 attributable deaths), this decreases strongly in the decarbonization pathways. Coal as a contributor to health impacts is almost eliminated (39 000 attributable deaths in IND_NZE_CLE, 23 000 in IND_NZE_MFR 2050). Remaining sources are mostly not directly related to fuel combustion. Industrial process emissions and emissions from agriculture are major contributors.

### 3.3. Effects of individual measures

In this section we analyze the sectoral policies taken in the mitigation scenarios. We use an aggregation similar to that applied in the UNEP/CCAC Assessment on solutions for Asia and the Pacific (UNEP 2019), which identified key measures for improving air quality in the region. Out of the full set of 25 measures in UNEP (2019), we selected a subset which seems of particular relevance for China and delivers air quality improvements. A detailed description of the measures is given in table S1 in the supplementary information.

Several of the mitigation measures analyzed here have effects on both air pollution as well as greenhouse gas emissions because they tackle common sources. It is of interest to look at 2030 and 2050 to see both the shorter as well as the longer time scale of policy action.

Several policies with co-benefits are already part of the Baseline, i.e. committed under current legislation and plans, such as transport electrification.

**Figure 3.**
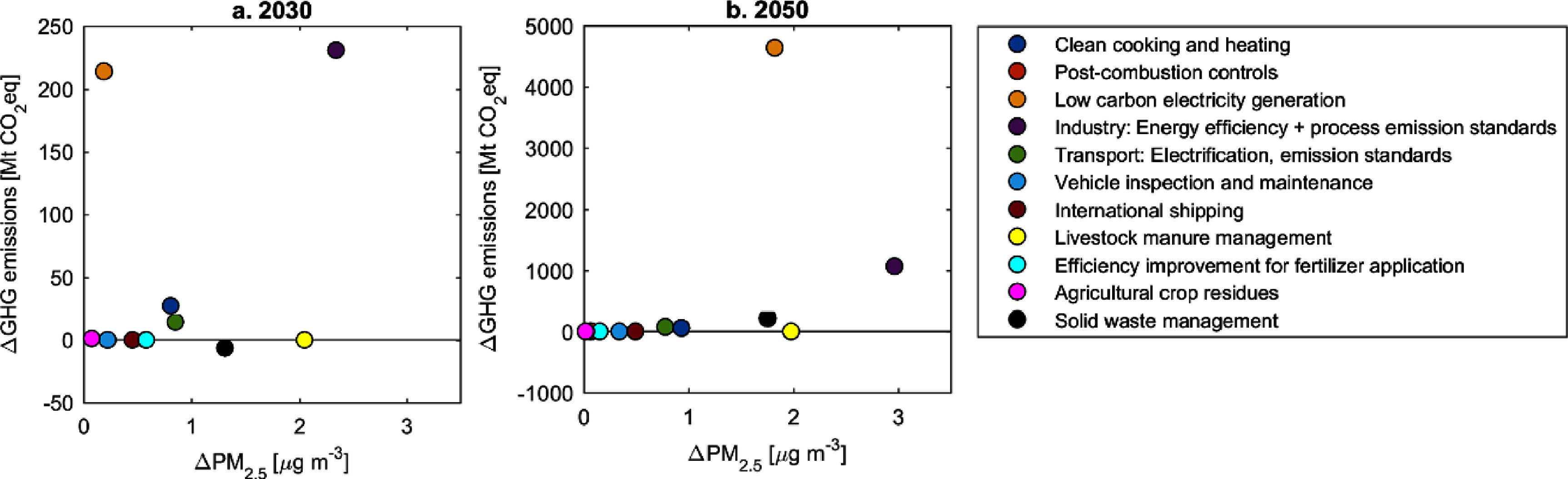
Co-benefits of measures for total China beyond the Baseline in 2030 (a) and 2050 (b).

Additional co-benefits of measures going beyond the Baseline are shown in figure 3 (numerical values in table A7), which shows for the whole of China the effects of measures on country-wide population weighted mean PM_2.5_ concentrations compared to the changes in GHG emissions (CO_2_ and CH_4_) they bring about. Such diagrams can be used to compare measures in terms of their potential to address both issues at the same time. The highest co-benefits between air pollution and GHG mitigation are reaped in measures in the upper right corner of the diagram. In 2030, industrial sources have the highest potential: implementing both energy efficiency and process emission standards to the maximum extent possible would improve mean PM_2.5_ concentrations by 2.3 *µ*gm^−3^ in 2030 (3.0 *µ*gm^−3^ in 2050) and at the same time reduce GHG emissions by 230 Mt CO_2_-eq (1067 Mt in 2050). Eliminating the remaining coal in the household sector, in conjunction with emission controls on open barbeques (measure ‘Clean cooking and heating’) has a potential for co-benefits in 2030 (0.8 *µ*gm^−3^ and 27 Mt CO_2_-eq saved), accelerating a transition which is assumed to happen at a longer time scale otherwise; in 2050 the transition is assumed to be completed already in the Baseline. Introducing more renewable fuels in the power sector (measure ‘Low carbon electricity generation’) will be inevitable for achieving the decarbonization pledges but it has very limited co-benefits for air pollution in 2030 thanks to already tight emission standards.

In 2050 the potential for co-benefits is dominated by two measures: replacement of coal in power generation with renewables and CCS plants, and energy efficiency improvements in industrial production, combined with stringent process emission standards. Other measures, such as agricultural manure management and improved waste management, will be important for PM_2.5_ concentration reductions, but the potential for GHG emission reductions from power and industry alone are an order of magnitude larger than any other measure.

### 3.4. Focus on Beijing

**Figure 4.**
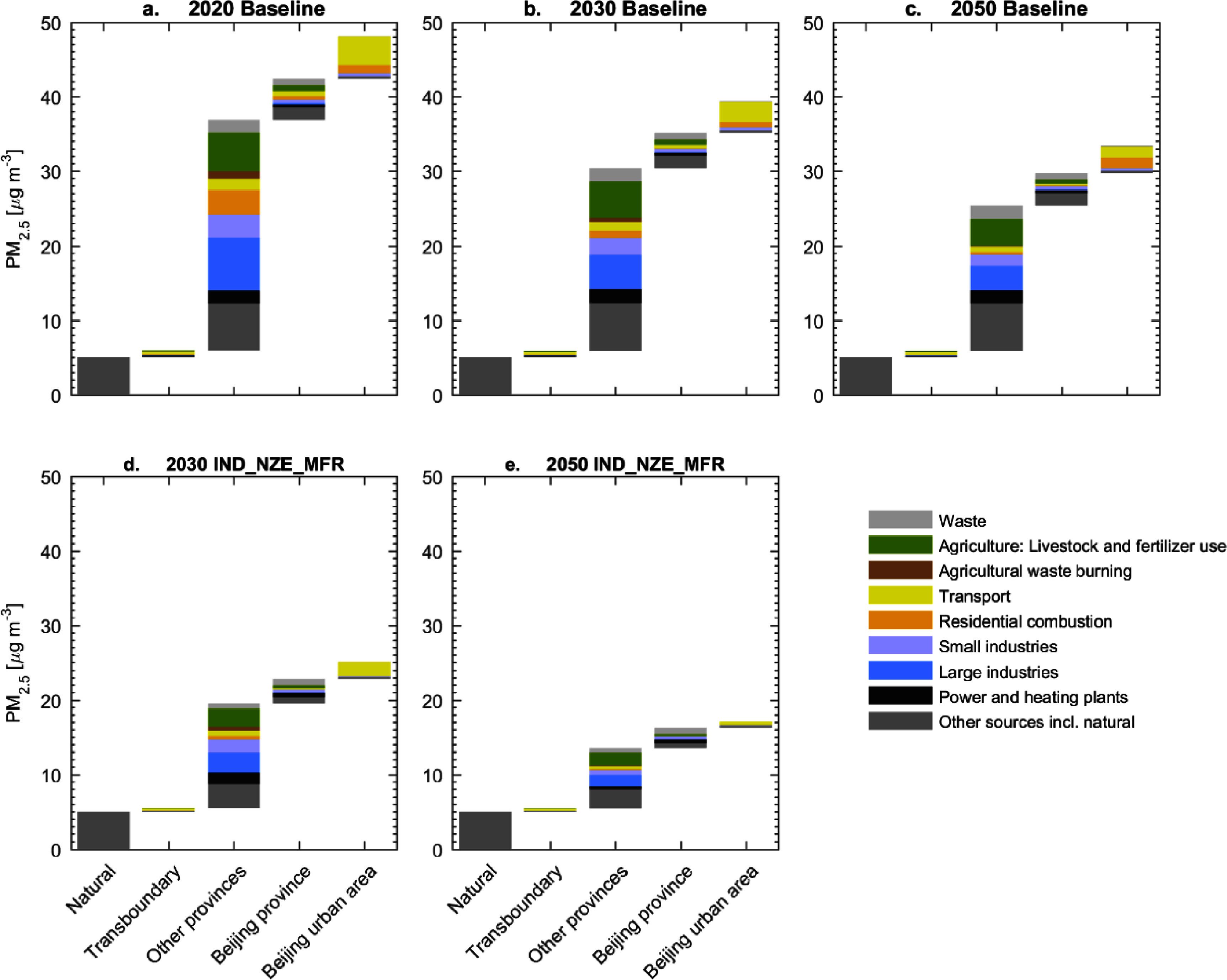
Ambient PM_2.5_ concentrations modelled in the city center of Beijing in different scenarios and years.

A megacity like Beijing faces specific issues for improving its air quality. In order to assess the relevance of individual mitigation options for Beijing, it is important to first understand the source contributions in terms of sectors and regions. Figure 4 (numerical results in table A8) shows the estimated contributions to ambient PM_2.5_ in central Beijing in different scenario years, disaggregated by spatial origin (Beijing urban area, Beijing whole, rest of China, other countries, natural sources) and economic sectors. Though it has been demonstrated before that megacities are often not responsible for the major share of ambient pollution they experience as they are influenced by inflow from surrounding areas (Amann *et al* 2017), it is remarkable how small the estimated contribution from Beijing to its own pollution is: In 2020 (figure 4(a)), 11.2 *µ*gm^−3^ of total PM_2.5_ in Beijing originate from the city, out of which 5.7 *µ*gm^−3^ from the urban area of Beijing. This is a result of successful control or elimination of many sources of local pollution in Beijing, particularly the relocation of heavy industry to surrounding provinces, and the switch to clean household fuels. Few years ago, residential coal heating was still contributing severely to ambient PM_2.5_ (Liu *et al* 2019). The largest remaining source in the city is road traffic, though its contribution in absolute terms is limited (3.8 *µ*gm^−3^ local contribution in the city center grid cell). Note that the atmospheric calculations are done at a resolution of 0.1° × 0.1° or roughly 10 × 10 km, which is sufficient to represent urban background levels but not local pollution hot spots.

The majority of pollution (64%) is transported into Beijing from its neighboring provinces, mostly from Hebei and Tianjin. Other studies (Wang *et al* 2024, Yin *et al* 2025) have pointed to substantial transboundary contributions to ambient PM_2.5_ in Beijing. The GAINS estimate is particularly high; however, a direct comparison is difficult due to different methodologies, emission inventories, and/or modelling periods. Significant contributions remain from industrial production (10.2 *µ*gm^−3^), largely fueled by coal. Also agriculture plays a big role as a precursor of secondary inorganic aerosols (5.3 *µ*gm^−3^). The weight of NH_3_ emissions versus the other secondary inorganic aerosol precursors SO_2_ and NO_x_ in this source attribution reflects the sensitivity of ambient concentrations to the respective emissions.

Unlike the strong decreases in ambient PM_2.5_ experienced in recent years, only moderate further improvement of the situation is foreseen under the Baseline scenario. With combustion emissions from industry and power absent in Beijing and already well controlled in surrounding provinces, the potential for fast decreases in these sectors has already been exploited.

Consequently, decarbonization steps in Beijing alone, as in the IND scenario, have only small additional benefits to ambient air quality because the sources affected by the decarbonization measures are already well controlled. In terms of city-mean concentrations, the reduction in the IND scenario compared to the Baseline is 0.8 *µ*gm^−3^ in 2030 and 1.6 *µ*gm^−3^ in 2050 (see figure S6 in the SI).

The weight of contributions from surrounding provinces remains high in the Baseline in 2030 (62%) and 2050 (58%). Contributions from industry remain significant in the future but these are mainly process related emissions, not combustion. Road traffic still contributes a few *µ*gm^−3^, mainly in the city and from non-exhaust emissions. Further switches out of coal in the residential sector marginalizes the role of heating and cooking. The remaining sources are largely non-energy related: agriculture and small diverse sources (‘other sources’) play a significant role. The IND_NZE_MFR scenario, shown in figures 4(d) and (e), reduces the contribution from Beijing’s surrounding provinces to less than half of the baseline situation, which is key to achieving strong concentration reductions in Beijing.

**Figure 5.**
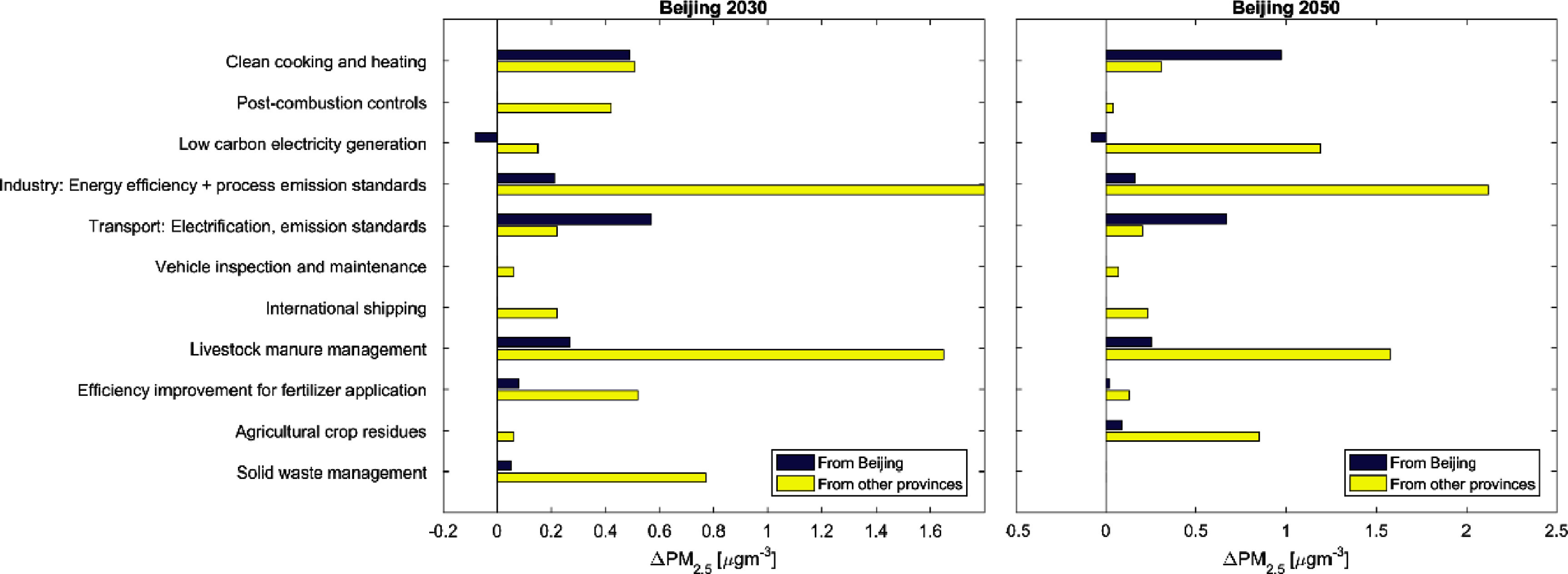
PM_2.5_ concentration changes in Beijing (population-weighted mean) expected from different sectoral measures applied on top of the Baseline in 2030 (a) and 2050 (b), distinguishing contributions from inside the province and from other provinces.

Figure 5 (numerical values in table A9) shows the expected improvement of ambient PM_2.5_ concentration levels in Beijing from individual sectoral measures when fully implemented in 2030 and 2050, compared to the Baseline situation in the respective year. Changes are broken down by contributions from Beijing province and surrounding provinces.

The largest concentration changes (2.5 *µ*gm^−3^ population-weighted mean PM_2.5_ in Beijing province in 2030; 2.0 *µ*gm^−3^ in 2050) are expected from measures in the agriculture sector, mostly from more efficient management of manure from livestock to reduce NH_3_ emissions during manure storage and application (1.8–1.9 *µ*gm^−3^ in either year) but also from improvements in nitrogen fertilizer application (reduction of mineral fertilizer use, replacement of urea). The residential sector continues to have a potential for concentration improvement by further advancing the transition to clean heating and cooking, in conjunction with measures to reduce heating energy demand through building insulation. Altogether, this measure can reduce PM_2.5_ in Beijing below Baseline levels by about 1.0 *µ*gm^−3^ (2030) to 1.3 *µ*gm^−3^ (2050), partly due to inflow of pollution from Hebei and Tianjin but also from rural parts of Beijing province itself, where a part of the population is still relying on solid fuel stoves (Meng *et al* 2019).

Another large potential (2.0–2.3 *µ*gm^−3^) lies in efficiency improvements and industrial process emission standards which could be implemented in the large production facilities in Hebei province. Post-combustion controls, i.e. flue gas cleaning from PM, SO_2_ and NO_x_, on the other hand have a much smaller additional potential. These controls have contributed to a large degree to the improvements in air quality after 2010 (Zhang *et al* 2019).

The highest potentials for further improving Beijing’s air quality from measures taken inside the city lie in the full electrification of road transport and concurrent strict emission standards on remaining internal combustion engine vehicles. Since China has already committed to a transition of road transport towards electric vehicles which is reflected in the Baseline scenario, these additional potentials concern mostly trucks or demand side measures like congestion charging.

## 4. Discussion and conclusions

The past decade has seen strong improvements in air quality in Beijing and other Chinese cities. As shown in this study, the next steps towards clean air will require considerably higher efforts, as many large sources of PM_2.5_ have already been controlled. Nonetheless, additional potential for mitigation clearly exists and will be partly harvested by existing legislation. Significant additional potentials for air pollution reductions are, for example, in industrial production and agriculture. Several of the measures reduce both PM_2.5_ and GHG emissions and therefore offer a clear potential for co-benefits. It is important to note, however, that decarbonization action alone will not solve the air pollution problem. It is clearly part of the solution, but in addition also policies targeting only air pollution will be needed in order to achieve a substantial reduction of ambient PM_2.5_.

With China’s current national ambient air quality standard of 35 *µ*gm^−3^ annual mean within reach, China has recently tightened this standard to 25 *µ*gm^−3^ by 2035 under the Beautiful China Policy released in January 2024, equivalent to the WHO interim target 2; according to the model calculations done here, reaching this target seems feasible after 2030 but only with additional efforts.

Improvements of air quality will be even more challenging for the megacity of Beijing since large industry is absent and residential heating has largely moved away from coal. Beijing still has a potential to reduce emissions by promoting the electrification of the residential and transportation sectors, as well as by strengthening the low-carbon energy codes for both existing and new buildings. In addition, we found that future reductions of Beijing’s ambient PM_2.5_ concentrations will largely depend on action in the surrounding provinces. A continuation of the integrated pollution management across the Jing-Jin-Ji priority area will thus be necessary. For example, the following policies for Hebei would significantly improve Beijing’s air quality due to transboundary effects: (a) Promote retrofitting technologies and waste heat utilization to improve energy and resource efficiency in the short term. (b) Accelerate the introduction of low-carbon process technologies in the steel, flat glass, and cement industries. (c) Improve the power transmission and distribution system with energy storage, essential for high electrification of end-use systems. (d) Build a carbon transportation and storage system in Hebei to support the acceleration of CCS deployment in the steel and cement sectors. (e) Strengthen collaboration across regional governments. Given the substantial role of agricultural NH_3_ emissions for ambient PM_2.5_ in Beijing, improved manure management and reduced mineral fertilizer use are priority measures in the non-energy sectors.

As with any model-based study, uncertainties exist in the results beyond the statistical uncertainties for mortality quantified in section 3.2. A full uncertainty analysis for all components of the model is beyond the scope of the study. While the measures discussed in section 3 cover most important sectors, some sources are not explicitly addressed and offer additional potential. For example, the representation of VOC emission sectors was considered not detailed enough in the GAINS model to analyze the potential for reduction measures. VOCs may form an important source of secondary organic aerosol in China (Liang *et al* 2023). Other sources like smoking, road dust resuspension, barbeques, ammonia emissions from sewage or sweat are not well understood and rather uncertain in the model and thus no specific measures are proposed for these. However, with other emissions well controlled, these sources may become important in the future. Furthermore, our analysis does not assume major behavioral changes such as diet shifts or a strong shift in transportation modes to walking, cycling and public transport. Both of these more systematic transformations would have health benefits not only from air pollution but also by reducing dietary risks (Willett *et al* 2019, Hamilton *et al* 2021), or more active life style (Lu *et al* 2022), while at the same time reducing GHG emissions.

Limitations exist also regarding the methodology applied in our study. When analyzing the effects of deep emission cuts on ambient PM_2.5_ concentrations, the GAINS model is rather conservative due to its use of linear atmospheric coefficients which approximate the responses well for small and moderate perturbations of emissions around the baseline, but do not represent effects of non-linear chemistry in secondary aerosol formation. The use of constant meteorological conditions in the future excludes a quantification of possible effects of changed meteorology. Also the assumption of time-independent emission factors is rather conservative and does not take into account possible future improvements in abatement technologies.

While we analyze potentials from sectoral measures for reduction of PM_2.5_ concentrations, we do not quantify costs of the measures and thus do not suggest a ranking in terms of cost effectiveness. Costs for technical air pollution control measures are quantified in the GAINS model, but costs for energy system transformation are not and therefore an overall cost effectiveness analysis is outside the scope of this paper. Such an analysis would be further complicated by the fact that decarbonization measures are taken primarily for other objectives than only air quality.

Although the analysis in this study was done specifically for Beijing and China, the GAINS model itself has a global coverage and, provided that energy projections for a specific region are available, similar analysis can be undertaken for other countries and cities.

## Data Availability

All data that support the findings of this study are included within the article (and any supplementary files).
